# Extended NICUs or specialized pediatric networks? The need to reinforce centralized, multidisciplinary care – a comment on Decembrino et al.

**DOI:** 10.1186/s13052-025-02170-w

**Published:** 2026-01-07

**Authors:** Giacomo Brisca, Pablo Mauricio Ingelmo, Anna Camporesi, Matteo Di Nardo, Andrea Dato, Zaccaria Ricci, Leonardo Bussolin, Giorgio Conti, Simonetta Tesoro, Carmelo Minardi, Geremia Zito Marinosci, Andrea Wolfler, Andrea Moscatelli

**Affiliations:** 1 Neonatal and Pediatric Intensive Care Unit, Intermediate Care Unit, Department of Emergency Medicine, Anesthesia, and Critical Care, IRCCS Istituto Giannina Gaslini, via Gerolamo Gaslini 5, 16147 Genova, Italy; 2 Pediatric Pain Treatment and Palliative Medicine Service, Department of Medicine and Surgery, Fondazione IRCCS San Gerardo dei Tintori, Milan Bicocca University, Monza, Milan, Italy; 3 Department of Pediatric Anesthesia and Intensive Care Unit, Buzzi Children’s Hospital, Milan, Italy; 4 Pediatric Intensive Care Unit, IRCCS Bambino Gesù Children’s Hospital, IRCCS, Rome, Italy; 5 Pediatric Intensive Care Unit, Meyer Children’s Hospital, IRCCS, Florence, Italy; 6 Department of Health Science, Section of Anesthesia and Intensive Care, University of Florence, Florence, Italy; 7 Italian Society of Pediatric and Neonatal Anesthesia and Intensive Care (SARNePI), Milan, Italy; 8 Sacred Heart Catholic University, Fondazione Policlinico Universitario Gemelli IRCCS, Rome, Italy; 9 Division of Anesthesia, Analgesia, and Intensive Care, Santa Maria della, Misericordia University Hospital, Perugia, Italy; 10 Department of Anesthesiology, Azienda Ospedaliero Universitaria Policlinico-San Marco, University of Catania, Catania, Italy; 11 Pediatric Critical Care Unit, Santobono-Pausilipon Children’s Hospital, Naples, Italy

## Abstract

The recent article by Decembrino et al. highlights the burden of RSV-related bronchiolitis admissions to Italian Neonatal Intensive Care Units (NICUs) during the 2021 season and proposes extending NICU roles to compensate for the national shortage of Pediatric Intensive Care Unit (PICU) beds. While acknowledging the urgent problem, we argue that such a strategy risks fragmenting pediatric critical care. Evidence consistently demonstrates that critically ill children achieve better outcomes in high-volume, specialized centers equipped with multidisciplinary expertise, resources, and continuous training. Italy currently counts only 273 PICU beds, corresponding to one per 35,856 children, far below European standards and with significant regional disparities—most notably in Southern Italy and Sardinia. International data indicate that higher patient volumes are associated with improved survival, supporting the consolidation of PICUs within sustainable hub-and-spoke networks. Regional initiatives, such as the Ligurian model integrating a central hub with 24/7 pediatric transport, demonstrate the feasibility of this approach. Future planning should focus on strengthening national referral systems, enhancing transport capabilities, and consolidating pediatric intensive care. With RSV prevention strategies evolving, Italy must build a resilient and flexible system, ensuring that all critically ill children have timely access to specialized, high-quality care.

To the Editor,

We read with great interest the article by Decembrino et al. [1], which describes the burden of RSV-related bronchiolitis admissions to Italian Neonatal Intensive Care Units (NICUs) during the 2021 epidemic season, and proposes the concept of “Extended NICUs” to address the national shortage of Pediatric Intensive Care Unit (PICU) beds.

While we share the authors’ concern regarding the limited PICU capacity in Italy, we believe that the proposed solution—broadening the role of NICUs to care for toddlers and critically ill infants—may lead to unintended fragmentation of pediatric care.

Critically ill children benefit from care in environments equipped with multidisciplinary pediatric expertise, including pediatric anesthesiology, surgery, subspecialties, and staff experienced in the physiological and psychological complexity of infants and toddlers. These elements are challenging to replicate across multiple smaller units, particularly in facilities with low pediatric patient volumes. Consolidating care in high-volume, specialized centers ensures better outcomes, continuity of care, and appropriate resource utilization.

## Current PICU capacity in Italy

European standards [2] suggest one PICU bed for each 20,000–30,000 children. As outlined by Minardi et al. [3], Italy currently counts only 273 PICU beds for a pediatric population of 9,788,622 children aged 1–18 years, corresponding to one bed per 35,856 children. Based on international standards, this translates into an estimated 44% national shortage. A breakdown by macro-area highlights marked regional disparities, with the most critical situation observed in Southern Italy, where the gap between available resources and patient needs is most evident. Particularly concerning is Sardinia, a geographically isolated region with no pediatric intensive care beds currently available (Table 1).

**Table 1:** PICU availability, annual admissions, and estimated bed shortages across Italian macro-areas

Italian Macro-areas	PICUs, *n*	PICUs admissions per year, median, *n* (IQR)	PICUs beds needed, *n*	PICUs beds available, *n*	PICUs beds shortage, %
North	8	301 (218.2-362.5)	222	128	42
Center	2	305 (296.5-313.5)	92	90	2
South	4	168.5 (135.2-230.2)	168	55	67
Total	14	774.5 (649.9-906.2)	482	273	44

## Evidence from international experience

International evidence demonstrates that simply multiplying small-volume units is neither clinically effective nor economically sustainable. Tilford et al. [4] analyzed 16 PICUs and found that for every additional 100 admissions, risk-adjusted mortality decreased (Odds Ratio [OR] 0.95, 95% Confidence Interval [CI]0.91–0.99) and length of stay was reduced. Similarly, Marcin et al. [5] demonstrated that higher volumes were generally associated with lower severity-adjusted mortality. Importantly, this relationship was non-linear (“reversed J-shaped”): mortality decreased with increasing volume up to a threshold, then rose again in the very highest-volume units. The lowest mortality was observed in PICUs with ~ 992–1,491 admissions per year, with the nadir around 1,250 admissions.

On this basis, we advocate the development of a sustainable Italian PICU network in which each unit serves an appropriately sized catchment area, ensuring patient volumes align with these standards. Exceptions may be necessary in geographically isolated areas, such as Sardinia, where a local PICU is justified despite lower volumes due to referral difficulties. Within such a network, PICUs should also be stratified according to level of specialization (e.g., community, tertiary, quaternary/specialized), as outlined by Frankel et al. [6].

## Policy framework and regional models

Both clinical and regulatory guidance support this vision. In particular, joint recommendations from the Italian Society of Anesthesia, Analgesia, Resuscitation and Intensive Care (SIAARTI) and the Italian Society of Neonatal and Pediatric Anesthesia and Intensive Care (SARNePI) [7] strongly support the development of regional pediatric care networks based on the hub-and-spoke model, in line with national legislation—such as Law no. 135 of August 7, 2012 [8], and the Ministerial Decree of April 2, 2015 (no. 70) [9]. These recommendations underscore the need to concentrate expertise and resources, reducing the current fragmentation of pediatric services across low-volume centers—a situation recognized as a latent systemic vulnerability.

In this spirit, the Liguria Region has attempted to implement a small-scale model of this very concept, connecting all regional pediatric units to a central hub, Giannina Gaslini Children’s Hospital [10]. A dedicated 24/7 pediatric transport system allows for the safe and timely centralization of all patients requiring advanced pediatric care. This system has already proven effective, particularly during the recent RSV epidemic seasons, in ensuring the coordinated and specialized management of severe bronchiolitis cases. Preliminary data, currently under review, suggest the adequacy and reliability of this integrated model. Similar solutions have been implemented in other Italian regions.

This pediatric referral system, developed by the Ligurian Government, aims to optimize pediatric care and ensure that every child receives timely treatment in the most suitable clinical setting. Although a virtuous example, it highlights the need to plan specialized pediatric services not only at the regional but also at the national level. This is particularly relevant for highly specialized fields, such as critical care, in the context of a steadily declining population. Unfortunately, beyond the cited policy documents and those listed in the references of Minardi et al. [11], no additional regulatory statements are currently available on this issue.

## Transport and referral networks

In the short term, simple organizational interventions could rapidly improve access to pediatric intensive care. Level III and IV PICUs should be formally designated as referral centers within the structured national network, coordinating both capacity and secondary transport of critically ill patients. Referral centers across northern, central, and southern Italy, if adequately supported, could ensure national coverage through ground, rotor, or fixed-wing transport. The Italian Air Force is institutionally responsible for life-saving ambulance flights and should be integrated into this network. Until bed availability is adequate, centralization should be guided by age and severity: the youngest and most critically ill should be referred to tertiary or quaternary centers; older patients may be managed in adult ICUs with pediatric support; and less severe infants may be safely cared for in NICUs, with telemedicine support from referral centers.

Structured pediatric transport systems are a key component of this model. In Italy, primary transports are managed by the Emergency Medical Services (the “so-called 118”), which may also manage secondary transfers. They will always need to stabilize pediatric patients on the scene, making it essential that they develop and retain pediatric competencies. Some referral centers have developed specialized transport systems capable of retrievals on a national basis, including ECMO (Extracorporeal Membrane Oxygenation) transports. When specialized services are not available, patients are transported by the referring institution, usually managed by anesthesia and intensive care specialists, pediatricians, or both in collaboration. Ideally, referral institutions should provide specialized transport services staffed by pediatric intensivists and PICU nurses, though complementary options (118 services, mixed teams) remain essential to address logistical or clinical constraints.

## Limitations of the “Extended NICU” model

Notably, the study by Decembrino et al. [1] highlights two particularly concerning findings. First, more than 30% of the NICUs included reported managing fewer than 10 bronchiolitis cases over a year, raising questions about the adequacy of experience and skill maintenance in such settings. Second, the authors acknowledge that many of these NICUs currently managing toddlers with bronchiolitis fall short of guideline-based standards. The limited use of high-flow nasal cannula (HFNC) and continuous positive airway pressure (CPAP), the frequent prescription of steroids, beta-2 agonists, and antibiotics, and the reported need for training in vascular access for older infants all point to gaps in pediatric-specific critical care skills. In contrast, such competencies—including appropriate respiratory support strategies, adherence to bronchiolitis treatment guidelines, and age-appropriate procedural expertise—are well-established and routinely practiced in specialized pediatric centers, where ongoing education and multidisciplinary collaboration are embedded in daily care.

Importantly, the challenges of pediatric intensive care extend far beyond bronchiolitis. While bronchiolitis has historically been a leading cause of PICU admission during the winter, this landscape is rapidly evolving. New preventive strategies, particularly extended immunoprophylaxis with nirsevimab, are already showing promising results. Real-world studies report ~ 80% reductions in RSV-related hospitalizations and PICU admissions, with lower odds of RSV hospitalization and PICU admission [12].

Therefore, a long-term healthcare planning model should not be built around a single disease, especially one undergoing epidemiological transformation. Rather, it should prioritize the development of resilient and flexible pediatric critical care systems, capable of addressing a wide range of variable, complex, and high-risk conditions in the most appropriate settings—centers with established infrastructure, multidisciplinary teams, and continuous training.

## Conclusions

In our view, especially in the context of a declining birth rate, the future of pediatric intensive care in Italy depends not on decentralization, but on the consolidation and networking of specialized services. With efficient transport systems in place, every child—regardless of where they live—can receive timely, high-quality, comprehensive care.

Finally, it is essential to remember that RSV infection is, in most cases, a self-limiting disease. The subset of patients requiring ICU admission is those at the highest risk of deterioration and thus need the possibility of rapid escalation of care. The cost of inappropriate care—be it an avoidable death or long-term disability—is far greater than the cost of the referral to a tertiary or quaternary care institution, for the patients, their families, and society as a whole.

## Data Availability

Not applicable.

## Abbreviations

NICU: Neonatal Intensive Care Unit
PICU: Pediatric Intensive Care Unit
HFNC: HighFlow Nasal Cannula
CPAP: Continuous Positive Airway Pressure
OR: Odds ratio
CI: Confidence Interval
ECMO: Extracorporeal Membrane Oxygenation
SIAARTI: Società Italiana di Anestesia, Analgesia, Rianimazione e Terapia Intensiva
SARNEPI: Società Italiana di Anestesia e Rianimazione Neonatale e Pediatrica
